# Discovery of Novel Focal Adhesion Kinase Inhibitors Using a Hybrid Protocol of Virtual Screening Approach Based on Multicomplex-Based Pharmacophore and Molecular Docking

**DOI:** 10.3390/ijms131215668

**Published:** 2012-11-23

**Authors:** Fengbo Wu, Ting Xu, Gu He, Liang Ouyang, Bo Han, Cheng Peng, Xiangrong Song, Mingli Xiang

**Affiliations:** 1 Department of Pharmacy and State Key Laboratory of Biotherapy, West China Hospital, Sichuan University, Chengdu 610041, China; E-Mails: wufengbo_@163.com (F.W.); hxclinphar@163.com (T.X.); ouyangliang@scu.edu.cn (L.O.); xiangrong_11@126.com (X.S.); tmkxiang@gmail.com (M.X.); 2 State Key Laboratory Breeding Base of Systematic research, Development and Utilization of Chinese Medicine, Chengdu University of Traditional Chinese Medicine, Chengdu 610041, China; E-Mail: hanbo@cdutcm.edu.cn

**Keywords:** pharmacophore, molecular docking, focal adhesion kinase, virtual screening

## Abstract

Focal adhesion kinase (FAK) is a tyrosine kinase that functions as a key orchestrator of signals leading to invasion and metastasis. In the current study, the multicomplex-based pharmacophore (MCBP)-guided method has been suggested to generate a comprehensive pharmacophore of FAK kinase based on seven crystal structures of FAK-inhibitor complexes. In this investigation, a hybrid protocol of virtual screening methods, comprising of pharmacophore model-based virtual screening (PB-VS) and docking-based virtual screening (DB-VS), is used for retrieving new FAK inhibitors from commercially available chemical databases. This hybrid virtual screening approach was then applied to screen several chemical databases, including the Specs (202,408 compounds) database. Thirty-five compounds were selected from the final hits and should be shifted to experimental studies. These results may provide important information for further research of novel FAK inhibitors.

## 1. Introduction

Focal adhesion kinase (FAK) is a 125-kDa non-receptor protein tyrosine kinase that was first identified in Src-transformed fibroblasts [1]. It is known that FAK is activated from integrin and growth factor receptors by auto-phosphorylation at Tyr397 [2], followed by subsequent activation of other functional phosphorylation sites to advance the signals to downstream pathways, such as AKT [3–5]. Based on these facts, FAK is thought to play a critical role in malignant behavior including proliferation, survival, and invasion [6,7]. FAK is overexpressed in many tumors, including those derived from the head and neck, colon, breast, prostate, liver, and thyroid [8–10]. Furthermore, FAK overexpression is highly correlated with an invasive phenotype in these tumors. Inhibition of FAK signaling by overexpression of dominant-negative fragments of FAK reduces invasion of glioblastomas [11] and ovarian cancer cells [12]. FAK therefore represents an important target for the development of anti-neoplastic and anti-metastatic drugs.

FAK play a critical role in the biological processes of cancer cells, so FAK has been proposed as a potential target in cancer therapy and small molecule inhibitors for use as potential cancer therapies have been developed [13]. Compounds: PF-573228; PF-562271; GSK-2256098 and TAE-226 have been recently generated by several groups. These compounds are ATP analogs and effectively inhibit the kinase activity of FAK [14,15]. However, there are still some problems in the development of drugs that obstruct FAK function. One of the possibilities for the development of an inhibitor of FAK is already well explained in a review by Nimwegen and Water [16]. The first possibility is inhibition of the kinase domain, thereby preventing the activation of downstream signaling cascades. But since FAK kinase activity itself may not be absolutely essential for its signaling functions, some problems have emerged. Lim *et al.* reported that FAK FERM-mediated nuclear localization of FAK promotes enhanced cell survival through the inhibition of tumor suppressor p53 independent of its kinase activity [17]. Another problem with the first possibility is the specificity of the kinase inhibitor, because kinase domains of a range of different proteins show a high degree of amino acid conservation in the catalytic domains [16].

As a part of the ongoing work in our research groups aimed at the search of selective FAK inhibitors, and our recent attempts to explore how to generate more accurate and reasonable structure-based pharmacophore models and virtual screening methods, the combined structure-based and ligand-based drug design strategy is useful to gain further insights into the molecular recognition patterns required for FAK protein binding, and for developing a multicomplex-based pharmacophore model that can be used for virtual screening to discover novel potential lead compounds. The multicomplex-based pharmacophore and virtual screening results can help us to predict the biological activities of the series compounds with a change in the chemical substitutions and to provide some useful references for the design of new FAK inhibitors. The theoretical results can offer some useful references for the design of new FAK inhibitors as anti-tumor drugs.

## 2. Result and Discussion

### 2.1. Generation and Validation of Multicomplex-Based Pharmacophore

**Figure 1:**
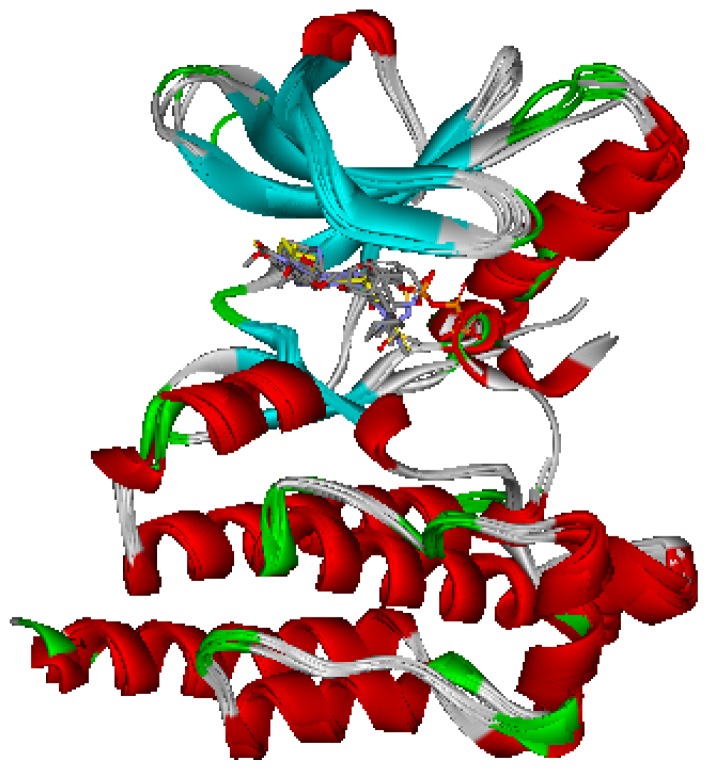
Superimposition of the seven FAK proteins.

**Figure 2:**
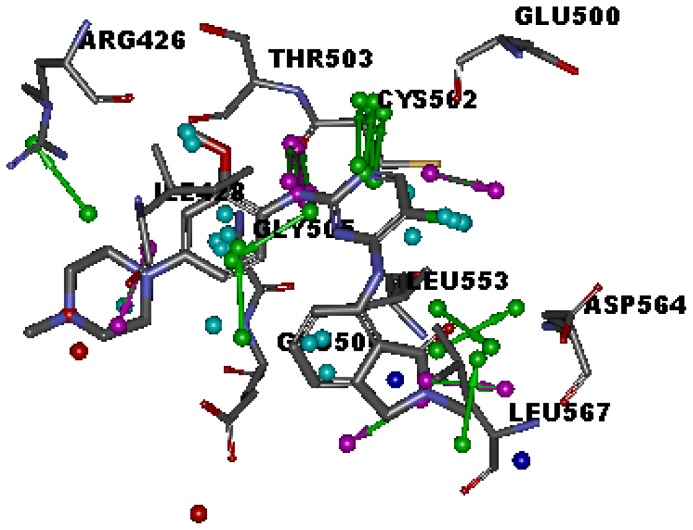
Specific regions of the ATP binding pocket of FAK.

**Figure 3:**
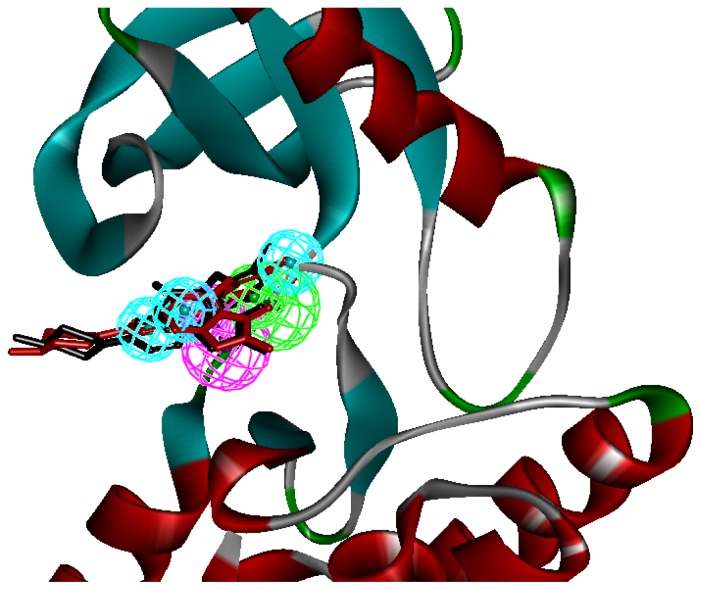
The mapping of multicomplex-based pharmcophore and the best mapping conformation (red bars) and the bound conformation (black bars) for the ligand to FAK are superimposed on the pharmacophore model. Screenshots were taken from Discovery Studio. Features of the pharmacophore models are color-coded as follows: hydrogen bond acceptor (HBA), green; hydrogen bond donor (HBD), violet; hydrophobic (HY), light blue.

Seven X-ray crystallography structures of FAK in complex with small molecular inhibitors were used to construct pharmacophore. Results of molecular superposition from the result based on Modeller [18] were reported below (Figure 1). The detected pharmacophore features as well as their statistical frequency, which measures how many complexes a given pharmacophore feature can be found in, were showed in Table 1. One can see that there were 15 pharmacophore features, including four hydrogen bond acceptor (A1–A4), four hydrogen bond donors (D1–D4), five hydrophobic features (H1–H5), one positive ionizable point and one negative ionizable point. In the 15 detected pharmacophore features, five features (A1, D1, H1, H2, and H3) were found to common in the seven complexes. It was believed that the pharmacophore features, which present in the complexes with a high probability, were likely to be more important than features exhibiting a low probability. For a full pharmacophore map, it was also important to include excluded volume features, which reflected potential steric restriction and corresponded to the positions that were inaccessible to any potential ligand. A comprehensive pharmacophore map and the ligand binding conformarion at the ATP site of FAK had been shown in Figure 2. The comprehensive pharmacophore map obtained initially was too restrictive and not suitable for the virtual screening since it contained a large number of chemical features and the fit of a molecule to such a pharmacophore was still out of reach for today’s state-of-the-art computational tools [19]. A correctly reduced pharmacophore model would be much more preferred in terms of practical application [20–22]. According to our experience, the top-ranked five features (A1, D1, H1, H2, and H3), would be more appropriate in practice, and consequently, they were selected from the comprehensive pharmacophore map and were merged to generate a multicomplex-based phamacophore (Figure 3). The difference of the chemical feature in this position between the ligand-based pharmacophore model and multicomplex-based pharmacophore was mainly due to the distinct methodologies that have been employed.

A reliable pharmacophore model may be used to determine the bioactive conformations of the ligands that share the same binding mode. The conformation selected for each compound, assumed as the bioactive conformation, corresponds to the conformation which best fits the pharmacophore. To verify whether the pharmacophore model finds the correct bioactive conformation, we applied the method to inhibitor TAF089, whose bioactive conformation is known from the co-crystal structure of FAK and the binding mode that is similar to the other derivatives. Thus, the X-ray crystal structure of FAK kinase (PDB code: 2JKO) was selected from the Protein Data Bank. The bound conformation of this inhibitor was respectively mapped onto the pharmacophore model using the *flexible* fitting method and the *best mapping only* option in the Ligand Pharmacophore Mapping protocol and meanwhile superimposed to the best mapping conformations (Figure 3). The RMSD value between the heavy atom positions of the bound and the best mapping conformation was 0.52 Å. The result showed that the pharmacophore model is capable of reproducing the bioactive conformation from the Protein Data Bank and support our choice for the bioactive conformation obtained from the best mapping conformation.

### 2.2. Parameter Setting and Scoring Function Selection for the Molecular Docking

Since docking parameters and scoring functions have important influence on the performance of molecular docking based virtual screening, we should carry out optimization for the docking parameters and scoring functions in advance. The Libdock module and Chemscore kinase in Discovery Studio were employed for DB-VS in the current study. The crystal structure of FAK complexed with TAF089 (PDB ID: 2JKO) was chosen as the reference receptor structure since it has the highest resolution (1.65 Å) among all the FAK crystal structures. We adjusted the docking parameters until the docked pose is as close as possible to the original crystallized structure in the ATP binding site of FAK. The finally optimized docking parameters mainly include: (1) the “number of hotspots” was set to 200; (2) the docking tolerance set to 0.20; (3) the “conformation method” was set to “FAST” and the other parameters were kept at the default settings. The RMSD value is 0.46 Å, which indicates that the docked pose is in accordance with the pose of the bound ligand in the crystal structure. For the selection of scoring functions, we chose a set of known FAK inhibitors whose IC_50_ values span a range of three orders. These inhibitors were docked into the ATP site of FAK, in which the docking parameters just optimized were used. Different scoring functions, including GoldScore, ChemScore, and a modified ChemScore—an optimized scoring function for the kinase-related docking (hereafter called Chemscore kinase)—were calculated. It was found that Chemscore kinase gave the best correlation coefficient. Therefore, Chemscore kinase will be used in the subsequent DB-VS study.

### 2.3. Database Screening

We have just finished the setup of these three procedures. Next, these methods will be applied to screen the Specs database (202,408 compounds). We used the three filters in the following order: PB-VS, DB-VS and drug-likeness filter, since a preliminary virtual screening test showed that PB-VS is faster than DB-VS in terms of the screening speed. This arrangement ensures that the first filter (PB-VS) is fast, while the successive one (DB-VS) is more time-consuming, but is applied only to a small subset of the entire database, which improves the screening efficiency. The flowchart of screening is shown in Figure 5. 37,654 compounds passed through the first filter PB-VS. These compounds were then filtered by DB-VS and 2386 compounds remained. The 2386 compounds were subsequently sorted according to their scoring function values calculated in DB-VS after the filtration of drug-likeness criterions. Finally, 35 compounds were visually chosen from the top hits. In order to be subsequently used to carry out the next assays, these compounds must satisfy the following criteria: (1) they have good interactions with the key residues in the active site of FAK, such as CYS502, THR503, GLY505 and LEU553 as well as residues in the hinge region (ARG426 to ILE428); (2) the Chemscore kinase value should be greater than 20; (3) these compounds should have scaffolds different from that of the known FAK inhibitors; (4) these compounds can be easily purchased.

**Figure 4:**
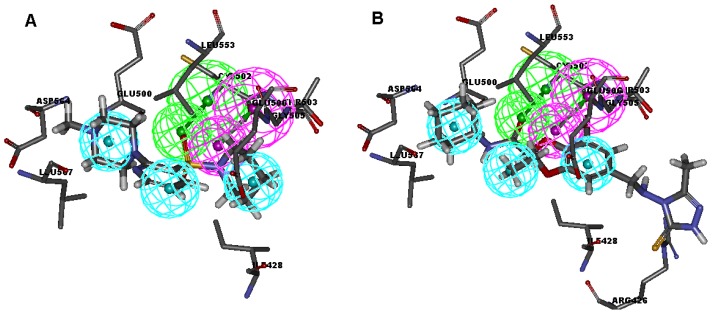
Multicomplex-based pharmacophore and docked binding models of the two representative compounds. (**A**) ZINC19815502; (**B**) ZINC00679655.

Among these hits, compounds ZINC19815502 and ZINC00679655, which are different in their chemical scaffolds, were identified as promising novel leads against FAK with high estimated IC_50_ value of 0.058 μM and 0.173 μM, respectively. They mapped all the features of multicomplex-based pharmacophore by choosing the best/flexible searching option (Figure 4), and the docked conformations with key residues in the active site of FAK (Figure 4). As they also satisfied all the drug-like properties, the two lead compounds would be focalized for further refining and optimizing to discover novel inhibitors with potent activity against FAK.

## 3. Computational Methods

### 3.1. Generation of Multicomplex-Based Pharmacophore Models

A set of seven crystal structures of FAK in complex with diverse ligands (Table 2) were obtained from the Protein Data Bank (PDB) [23]. Water molecules in ligand-binding sites have been reported to play a crucial role in mediating the interactions between FAK and its ligands, and they can provide useful information for the process of pharmacophore construction [24]. Therefore, all the water molecules in the crystal structures were retained. The coordinates of seven FAK-ligand X-ray crystal structures were transformed into a common reference frame by using “Multiple Structure Alignment (Modeller)” module within the Discovery Studio (DS) [25].

**Figure 5:**
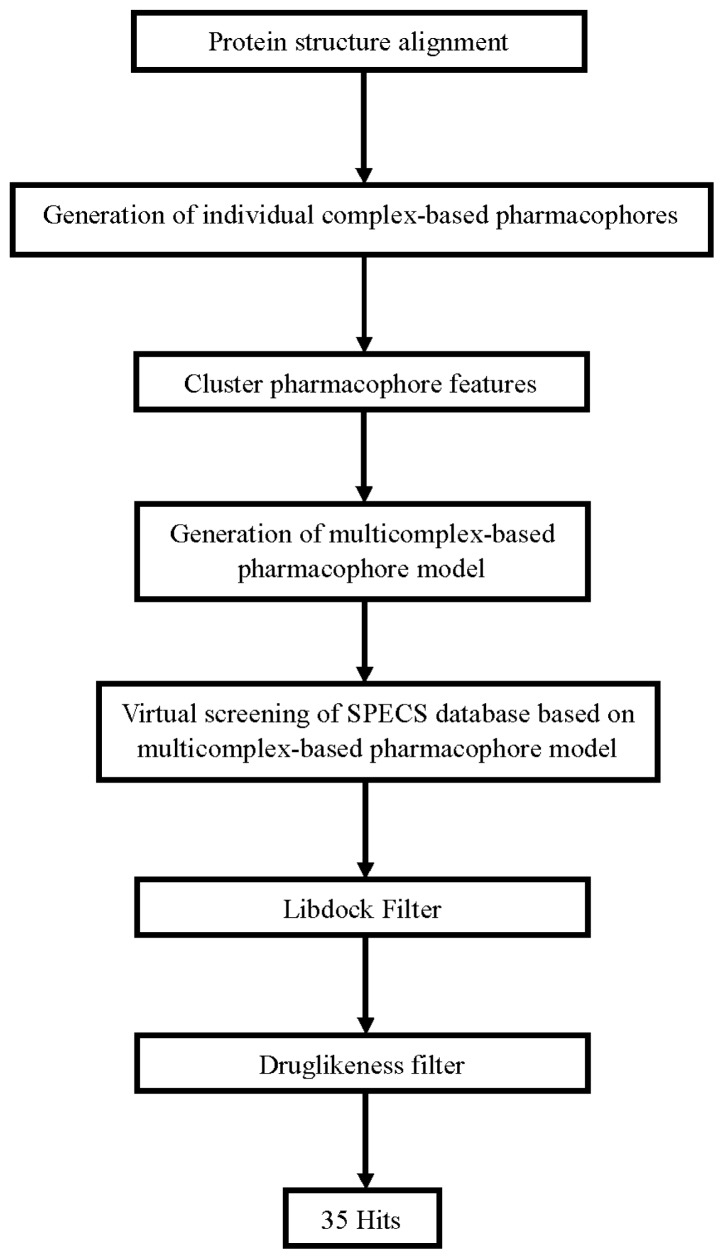
Steps of the generation of the multicomplex-based pharmacophore model and the hybrid virtual screening approach based on multicomplex-based pharmacophore model and molecular docking.

The whole process of generation and utilization of the multicomplex-based pharmacophore models were illustrated in Figure 5 and detailed as follows. The complex-based pharmacophore generation module in DS was used to generate seven individual complex-based pharmacophore models based on the previously aligned structures. For the purposes of creating a multicomplex-based pharmacophore model, all the pharmacophore features identified by DS were clustered according to their interaction pattern with the receptor. The cluster centers were identified using the Discovery Studio [25]. The model obtained was further refined by the modification of the constraint tolerance of the spheres in accordance with the default values of Catalyst modules in Discovery Studio.

### 3.2. Pharmacophore-Based Virtual Screening

Virtual screening of chemical databases may facilitate finding novel lead compounds suitable for further research. Compared to other *de novo* design methods, virtual screening has the advantage that the hit compounds can be easily obtained from commercial sources for biological activity assay. [26] In the present study, we performed all database searching experiments using “Best” conformation generation option and “Flexible” fitting method option in the “pharmacophore” protocols of Discovery Studio software. The molecules in the SPECS database ensemble in the ZINC database which fit on all the features of the pharmacophore model were retained as hits. [27] Geometric fit values were calculated for every hit compound based on how well the chemical substructures mapped on to the pharmacophore features. The criterion for screening for further validation was high fit values, which indicate good matches. Hit compounds with the fit value over 3.0 were selected and evaluated for their drug-likeness properties using Lipinski’s rule of five and ADME/T (Absorption, Distribution, Metabolism, Excretion and Toxicity) filters in Discovery Studio.

### 3.3. Molecular Docking Study

All the molecular docking studies were carried out by Libdock module in Discovery Studio, and the CHARMm force field was used. The crystal structure (PDB entry 2JKO) of the kinase domain of FAK bound to TAF089 was taken as the receptor structure. The binding site was defined as a sphere containing the residues that stay within 10 Å from the co-ligand, which cover the ATP-binding region and hinge region at the active site.

## 4. Conclusion

In conclusion, we utilized seven crystal structures of human FAK bound to small molecular inhibitors to generate a multicomplex-based pharmacophore. It has been validated that the multicomplex-based pharmacophore model was capable of predicting the bioactive conformations and molecular alignments of a wide variety of FAK inhibitors in the structurally diverse datasets.

This work conducted here had provided an approach to generate a multicomplex-based pharmacophore-guided virtual screening based on a set of crystal structures of protein–ligand complexes. And the multicomplex-based pharmacophore-guided virtual screening can be used for further discovery and to design more potent FAK inhibitors and to evaluate the newly engineered compounds in *de novo* design. The studies suggest that in the search of novel inhibitors, the multicomplex-based pharmacophore-guided virtual screening could be useful in getting the predictive models which may provide useful information required for proper understanding of the important structural and physicochemical features. Furthermore, a hybrid protocol of virtual screening methods including PB-VS and DB-VS has been introduced in the discovery of FAK inhibitors. We have established a pharmacophore model of FAK inhibitors for the PB-VS method. The docking parameters were also optimized in advance. Finally, the hybrid VS approach was applied to screen Specs chemical databases (202,408 compounds). Thirty-five compounds were selected from the final hits and should be shifted to the subsequent experimental studies.

## Figures and Tables

**Table 1:** Analysis and comparison of pharmacophore model features.

No.	Feature Name	ID	Count	Statistical frequency (%)	Structure-based Pharmacophore model	Related amino acid residues
1	HBA-F 1	A1	4	57	√	Cys 502
2	HBA-F 2	A2	2	28		
3	HBA-F 3	A3	2	28		
4	HBA-F 4	A4	1	14		
5	HBD 1	D1	4	57	√	Thr 503
6	HBD 2	D2	2	28		
7	HBD 3	D3	1	14		
8	HBD 4	D4	1	14		
9	Hydrophobic 1	H1	4	57	√	Ile 428, Gly 505
10	Hydrophobic 2	H2	4	57	√	Met 499, Leu 553
11	Hydrophobic 3	H3	3	43	√	Leu567
12	Hydrophobic 4	H4	2	28		
13	Hydrophobic 5	H5	1	14		
14	Negative Ionizable	Neg	1	14		
15	Positive Ionizable	Pos	1	5		

**Table 2:** List of seven FAK protein–ligand complexes used in the study.

No.	PDB	Resolution	Ligand	Release date
1	2ETM	2.30	7PY	2006-10-10
2	2IJM	2.19	ATP	2007-8-14
3	2JKK	2.00	BI9	2008-9-9
4	2JKM	2.31	BII	2008-9-9
5	2JKO	1.65	BIJ	2008-9-9
6	2JKQ	2.60	VG8	2008-9-9
7	3BZ3	2.20	YAM	2008-4-1
